# Supplementary material for: In-hospital cardiac arrest (IHCA): survival status and its determinants in Malaysian public healthcare
Source: PeerJ. 2025 Jul 4;13:e19509. doi: 10.7717/peerj.19509 (PMC12232926; doi:10.7717/peerj.19509)
Supplement: Supplemental Information 1 [file peerj-13-19509-s001.docx]

**Table_S1 Data Domains and Codebook for IHCA study**

**Demographic features, clinical features, and post arrest outcomes of IHCA patients**

| **Data Domain** | **Data Elements** |
| --- | --- |
| *Patient demographic features* |  |
| Demographic  Current hospital admission | Age, gender and race |
|  | Date and reason of admission |
| *Clinical features* |  |
| Resuscitation | Date, time, and duration of resuscitation (minutes)  Location of cardiac arrest (CA)  First documented cardiac arrest rhythm  With by-stander/without by-stander CA  Rescuer’s qualification: ALS certified and designation: house officer, medical officer, specialist Total doses of adrenaline used.  Total number of defibrillation shocks given  ROSC  Survival status |
| Post Cardiac Arrest Care | Location post-arrest care |
| *Post-arrest Outcomes* | CPC at discharge  Date of discharge  Methods used to assess CPC  Date and time of death  Cause of death |

**Code book for IHCA study**

| **Independent Variables** | **Code** | **Definition** | **Scale measurement** |
| --- | --- | --- | --- |
| Age | Age | As indicated in the respondent’s year of birth in the identification card | Continuous data measured in year |
| Gender | Sex | As indicated in the patient’s case note | 1= Male  2= Female |
| Ethnicity | Race | As indicated in the patient’s case note | 1= Malay  2= Chinese  3= Indian  4=Others |
| Reasons for Admission | ReasonAdmission | Reasons for Admission | 1= Elective Surgery  2= Emergency Surgery  3= Medical  4= Trauma  5= Others |
| Time of IHCA event occurs | Timing IHCA | Timing of the cardiac arrest occurred in relation to the office hour or non-office hour | 1= office hours  2= out of office hours |
| Location of IHCA event occurs | Arrest Location | Location of the cardiac arrest case | 1= Ward  2= Critical care area 3= Emergency Department  4= Others |
| First Documented Cardiac Rhythm | FirstDocumentedRhythm | First Documented Cardiac Rhythm | 1= Pulseless electrical activity  2= Ventricular fibrilation  3= Ventricular Tachycardia  4= Asystole |
| Arrest Witnessed | ArrestWitnessed | Cardiac Arrest Witness | 0= no 1=yes |
| Rescuer ALS-Trained | RescuerQualification | Rescuer ALS-Trained or not trained | 0= no 1= yes |
| Rescuer Designation | Rescuer | The medical staff that rescue the IHCA case | 1= Medical Officer  2=House Officer  3=Specialist  4=Others |
| Duration CPR | DurationMins | Duration of Cardiopulmonary Resuscitation | Continuous data measured in minutes |
|  |  |  |  |
| Adrenaline to CPR Ratio | AdrCPRRatio | Adrenaline to CPR Ratio | Continuous data constructed total duration of CPR divided by the total adrenaline dosage  1=<3min  2= 3-5min  3= >5min |
| Return of Spontaneous Circulation (ROSC) | ROSC-non-ROSC | Success Return of Spontaneous Circulation, or no ROSC | 0=non-ROSC 1=ROSC |
| Survival Status | Survival_Sta | Patients survive (alive)/not survived (death) | 0= Death  1=Survived/alive |
| Causes of Death | Causes_Death | Causes of death as per declared on certification | 1=CVD 2= Septicaemia 3= Cancer 4= Git/Hepato 5= Trauma 6=Organ Failure 7=Respiratory 8= Kidney  9= Others  10=pending postmortem  11= not doc |
| Time of Resuscitation to Discharged | Time TDF1 | Time of Resuscitation to Discharged | hours |
| Time from ROSC to Discharge | T3 | Time from ROSC to Discharge | hours |
| Patient status | Patient stat | Death/discharge/AOR | 1=Death, discharge  2=AOR |
| Rescuer ALS training duration | ALS_more_than_3years | Rescuer ALS training duration (<3years/>3years) | 0= no  1= yes |
| Post resus location | PRL | Post ROSC location care | 1= ED  2= Critical care  3= Ward |
| CPC at discharge | CPC_stat | Cerebral Performance Category (CPC) at discharge | 1= CPC 1  2= CPC 2  3= CPC 3  4= CPC 4 |

**Compliance Table Based on the 2018 Utstein IHCA Elements Consensus Definition**

Reference manuscript: (Nolan et al., 2019)

Cardiac Arrest and Cardiopulmonary Resuscitation Outcome Reports: Update of the Utstein Resuscitation Registry Template for In-Hospital Cardiac Arrest: A Consensus Report from a Task Force of the International Liaison Committee on Resuscitation (American. Resuscitation 144:166–177. DOI: 10.1016/j.resuscitation.2019.08.021.

| **Category** | **Element** | **Definition** | **Compliance Status** |
| --- | --- | --- | --- |
| Hospital - Core | Number of hospital admissions per calendar year | Total hospital admissions, including day cases but excluding outpatients and visitors. | Not in IHCA registry.  Data available in the Hospital Registration System |
|  | Number of treated in-hospital cardiac arrests per calendar year | Defined as cases where chest compressions and/or defibrillation were delivered. | Not in IHCA registry.  Data available in Record Office |
| Hospital - Supplemental | Total number of hospital deaths per calendar year | Number of deaths recorded in the hospital. | Not in IHCA registry.  Data available in Record Office |
|  | Hospital description | Includes details such as total number of beds, ICU beds, pediatric beds, availability of a 24/7 cardiac catheterization lab, and use of a rapid response system. | Not in IHCA registry.  Data available in Record Office |
| Patient - Core | Age | Date of birth in the format DD/MM/YYYY or MM/DD/YYYY, or marked as unknown. | Yes, data available in our IHCA registry |
|  | Sex | Male/Female/Unknown. | Yes, data available in our IHCA registry |
| Patient - Supplemental | Race | Defined based on national guidelines for race categories. | Yes, data available in our IHCA registry |
|  | Out-of-hospital cardiac arrest | Indicates whether the patient had a cardiac arrest before hospital admission (Yes/No/Unknown). | Not in IHCA registry.  Data available in patient’s folder/case note |
| Pre-Event Factors | Subject type | Categorizes the patient (e.g., outpatient, inpatient, visitor, etc.). | Yes, data available in our IHCA registry |
|  | Illness category | Categorizes illness as medical-cardiac, medical-noncardiac, surgical-cardiac, surgical-noncardiac, obstetric, trauma, or other (visitor/employee). | Yes, data available in our IHCA registry |
| Cardiac Arrest Process - Core | Date/time of event | The date and time when chest compressions or defibrillation (if applicable) were first required. | Yes, data available in our IHCA registry |
|  | Event location | The specific location within the hospital where the cardiac arrest occurred. | Yes, data available in our IHCA registry |
|  | Event witnessed | Indicates whether the arrest was seen or heard by another person or monitored. | Yes, data available in our IHCA registry |
|  | Resuscitation team called | Indicates whether a hospital-wide resuscitation response was activated. | Yes, data available in our IHCA registry |
|  | Monitored cardiac arrest | Whether ECG or other monitoring was in place at the time of the arrest. | Not in IHCA registry.  Data available in patient’s folder/case note |
|  | Initial rhythm | First documented cardiac rhythm when chest compressions or defibrillation were initiated. | Yes, data available in our IHCA registry |
|  | Chest compressions | Indicates whether chest compressions were administered. | Yes, data available in our IHCA registry |
|  | Automated external defibrillator (AED) used | Indicates whether an AED or a manual defibrillator in AED mode was applied. | Yes, data available in our IHCA registry |
| Post-resuscitation Process - Core | Targeted temperature management | Active therapy to achieve and maintain a target temperature for a defined period. | Not in IHCA registry.  Data available in patient’s folder/case note |
|  | Avoidance of pyrexia | Preventing fever (>38°C) within 72 hours post-arrest. | Yes, data available in our IHCA registry |
|  | Coronary angiography | Categorized into urgent (within 2 hours post-arrest) and delayed interventions. | Not in IHCA registry.  Data available in patient’s folder/case note |
|  | Attempted coronary reperfusion | Whether percutaneous coronary intervention (PCI) or thrombolysis was attempted. | Not in IHCA registry.  Data available in patient’s folder/case note |
| Outcome - Core | Any ROSC (Return of Spontaneous Circulation) | Defined as restoration of circulation without ongoing chest compressions. | Yes, data available in our IHCA registry |
|  | 30-day survival or survival to discharge | Indicates whether the patient was alive at hospital discharge or after 30 days. | Yes, data available in our IHCA registry |
|  | Neurological outcome at 30 days or hospital discharge | CPC (Cerebral Performance Category), PCPC (Pediatric Cerebral Performance Category), or mRS (Modified Rankin Scale) score recorded at 30 days or discharge. | Yes, data available in our IHCA registry |
|  | Organ donation | Whether the patient donated one or more organs (Yes/No/Unknown). | Data not available |
