# Supplementary material for: In-hospital cardiac arrest (IHCA): survival status and its determinants in Malaysian public healthcare
Source: PeerJ. 2025 Jul 4;13:e19509. doi: 10.7717/peerj.19509 (PMC12232926; doi:10.7717/peerj.19509)
Supplement: Supplemental Information 3 [file peerj-13-19509-s003.docx]

**Table S3 Cerebral Performance Category (CPC) Scale**

| **Category** | **Description characteristics** |
| --- | --- |
| CPC 1 | Normal (good cerebral performance)  Able to work and conduct a normal life while attentive and conscious.  Possibility of having minimal neurologic or psychologic problems (e.g., minor cranial nerve abnormalities, non-incapacitating hemiparesis, mild dysphasia). |
| CPC 2 | Moderate disability (disabled but independent)  Conscious and capable of performing independent daily tasks (such as dressing, using public transportation, and preparing food) or part-time job in a safe environment. |
| CPC 3 | Severe disability (conscious but disabled and dependent)  Conscious yet reliant on others for support daily (at a facility or at home with outstanding family effort) or at least has limited cognitive abilities.  Numerous neurologic dysfunctions fall under this group. |
| CPC 4 | Unconscious (coma or vegetative state)  Unconscious, no cognition, no awareness of surrounds. No verbal or psychological interaction with the environment. |
