# Supplementary figures and images for: In-hospital cardiac arrest (IHCA): survival status and its determinants in Malaysian public healthcare
Source: PeerJ. 2025 Jul 4;13:e19509. doi: 10.7717/peerj.19509 (PMC12232926; doi:10.7717/peerj.19509)

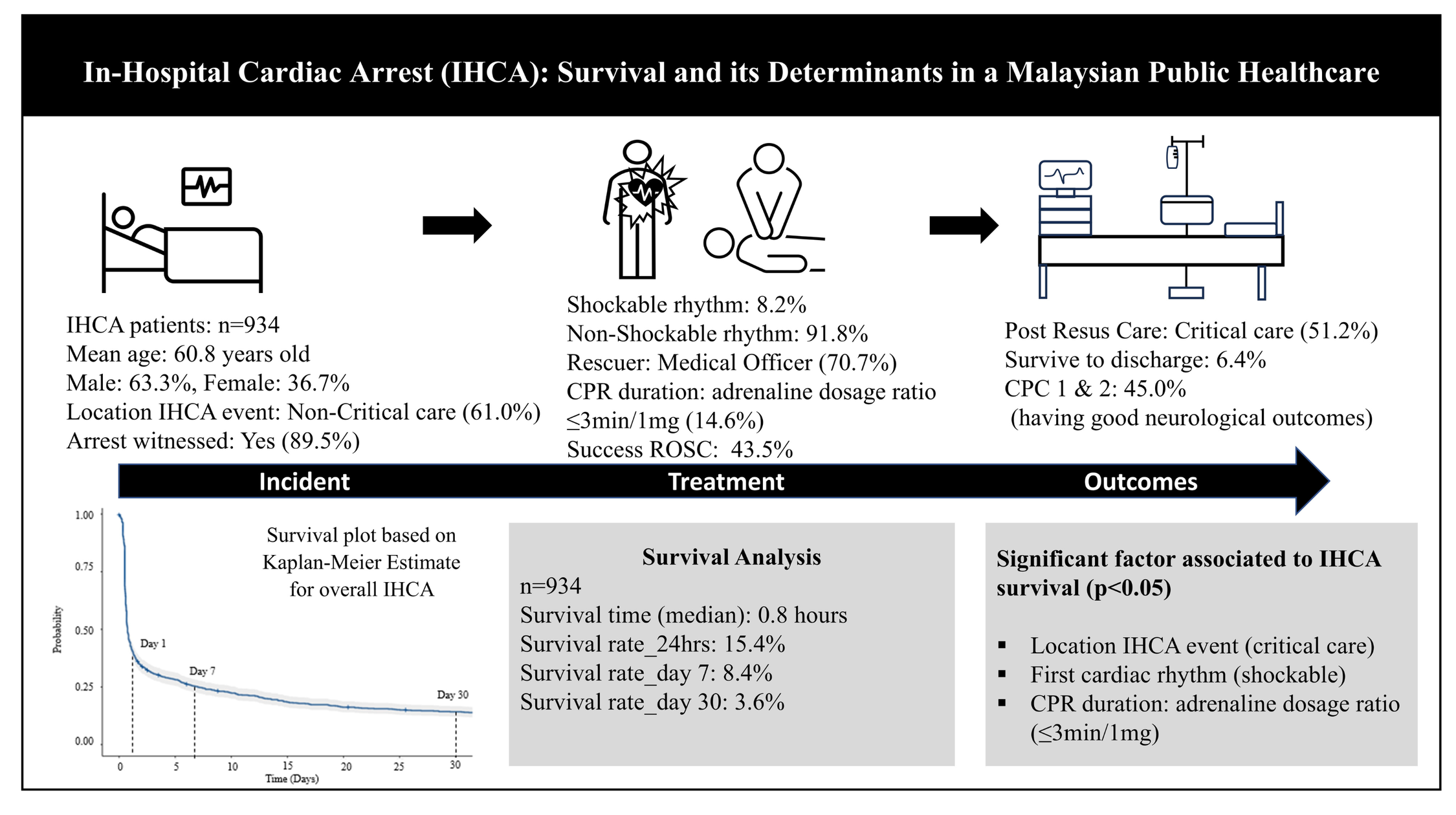

Supplement: Supplemental Information 5 [file peerj-13-19509-s005.png]
